# Lyme Carditis Manifesting as Wenckebach Heart Block

**DOI:** 10.7759/cureus.19251

**Published:** 2021-11-04

**Authors:** Iyad Aljadba, Krithika Suresh, Khandakar M Hussain

**Affiliations:** 1 Internal Medicine, Conemaugh Memorial Medical Center, Johnstown, USA

**Keywords:** lyme's disease, av block, wenckebach, bradycardia, atrio-ventricular block, lyme carditis, carditis, sinus pause, syncope

## Abstract

Lyme disease caused by *Borrelia burgdorferi* is a multisystem disease and can lead to Lyme carditis. The most common presentation of Lyme carditis is conduction system disturbances such as atrioventricular (AV) block. A 72-year-old male with a past medical history of gastroesophageal reflux disease (GERD) and prostate cancer presented with chest tightness, lightheadedness, and presyncope. During hospitalization, he developed bradycardia with heart rates ranging between 30 and 40 beats per minute and Wenckebach heart block. Lyme serology was consistent with a recent Lyme infection. He was treated with antibiotics and was eventually discharged home without requiring pacemaker implantation.

## Introduction

Lyme disease caused by the spirochete *Borrelia burgdorferi* is the most common vector-borne disease in the United States [1]. The causative spirochete is mainly transmitted by four species of Ixodes tick [2]. Early localized infection is manifested by the characteristic erythema migrans skin lesion, while early disseminated disease includes neuroborreliosis manifesting as meningoradiculoneuritis and Lyme carditis. The most common manifestation of late Lyme disease is Lyme arthritis [3].

## Case presentation

A 72-year-old male with a past medical history significant for non-obstructive chronic artery disease (CAD), gastroesophageal reflux disease (GERD), prostate cancer post-resection presented to the emergency department (ED) with left-sided chest tightness, lightheadedness, and presyncope. He also reported mild exertional shortness of breath. His initial vitals were stable with a heart rate (HR) of 65 beats per minute and blood pressure (BP) of 112/66 mmHg. Initial labs including complete blood count, metabolic panel (K: 4.2, Mg: 2.1, Ca: 9.1) initial troponin, and chest X-ray were largely unremarkable. Coronavirus disease 2019 (COVID-19) reverse transcriptase-polymerase chain reaction (RT-PCR) was negative. Initial electrocardiogram (EKG) at 6:17 PM showed normal sinus rhythm, rate of 65 beats per minute without ST-T wave changes. He was admitted to the floor for further evaluation and management. It was noted that the patient did have a recent exercise stress test (with myocardial perfusion scan) as an outpatient which was negative for ischemic changes. He also had a recent echocardiogram which showed a normal ejection fraction (EF) of 60% with mild mitral regurgitation. Orthostatic vitals were checked and were negative (lying BP: 100/55 mmHg, seating BP: 99/54 mmHg, and standing BP: 100/61 mmHg). Overnight, he was noted to develop symptomatic bradycardia with HR on the cardiac monitor between 30-40 beats per minute (bpm).

**Figure 1:**
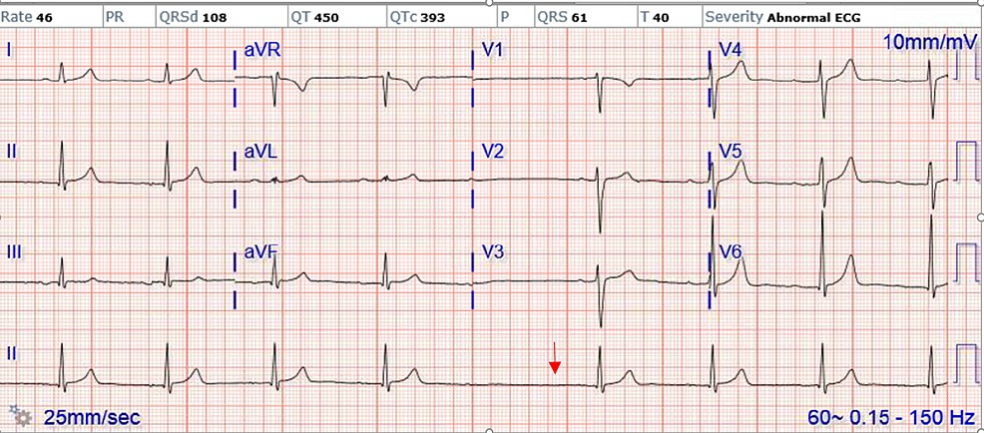
EKG showing Mobitz type 1 heart block aVR: augmented vector right; aVL: augmented vector left; aVF: augmented vector factor

**Figure 2:**
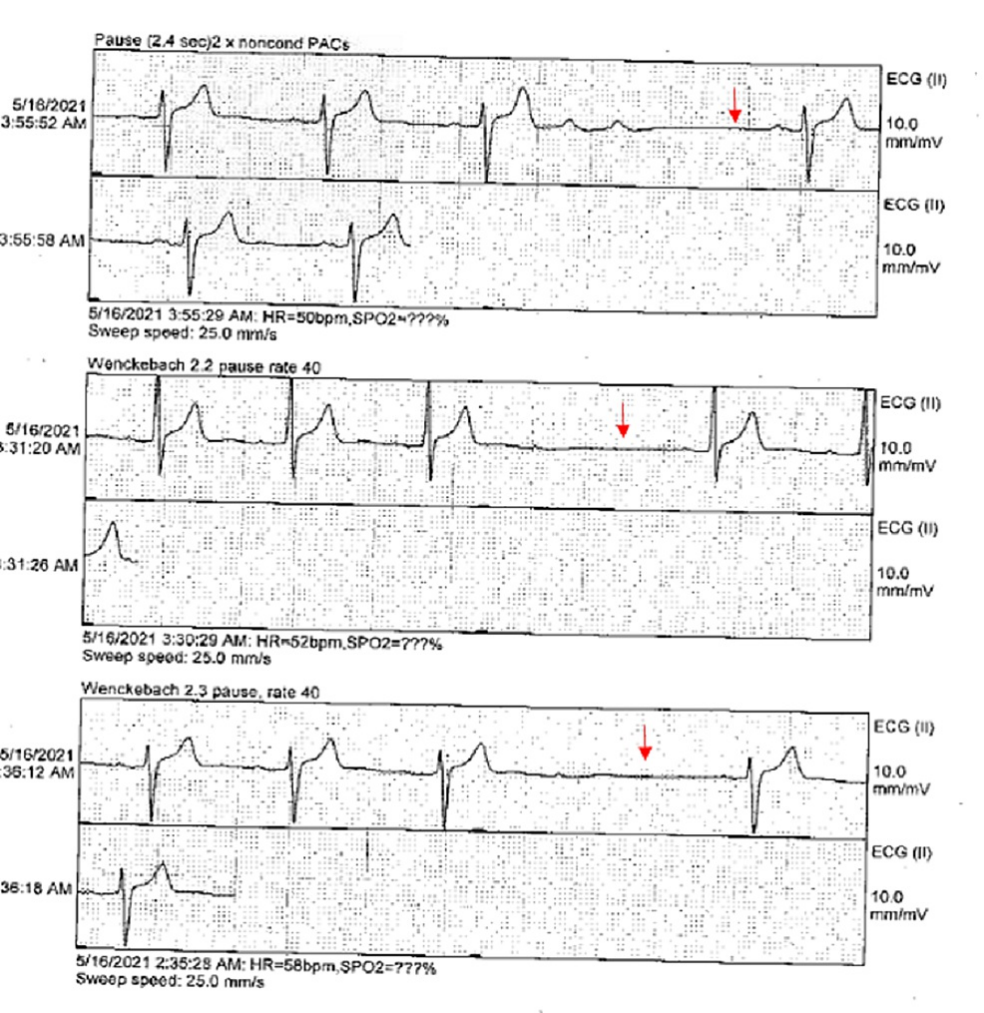
Telemetry showing Wenckebach heart block

**Figure 3:**
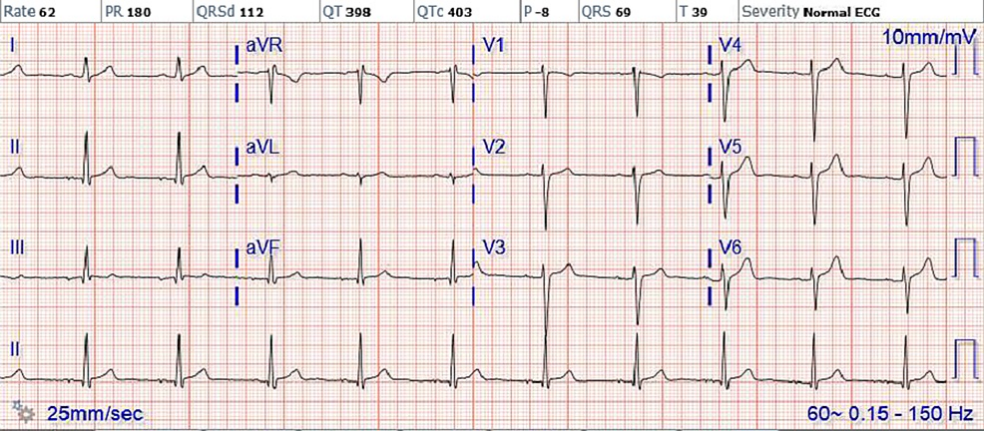
EKG showing normal sinus rhythm aVR: augmented vector right; aVL: augmented vector left; aVF: augmented vector factor

Repeat EKG obtained in the morning showed sinus bradycardia with Mobitz type 1 (Wenckebach) heart block and progressive prolonged PR interval (Figure 1). Telemetry also revealed Wenckebach's heart block (Figure 2). Lyme titers were ordered and came back reactive and Western blot was positive for IgG (10/10 bands); however, negative for IgM consistent with Lyme infection that occurred more than a month prior. He was treated with IV ceftriaxone 2 g every 24 hours with the resolution of his heart block and bradycardia after a total of seven days of IV antibiotics (Figure 3). He was eventually discharged home on doxycycline 100 mg p.o. for 21 days to complete 28 days total with recommendations to follow up with cardiology as an outpatient without needing pacemaker implantation.

## Discussion

Lyme carditis (LC) ranges between 1% and 10% of total Lyme disease cases, occurring within weeks to months of the onset of infection. It presents in the early disseminated stage, the second phase of the disease [4]. Clinical manifestations include syncope, lightheadedness, shortness of breath, palpitations, and/or chest pain. Atrioventricular (AV) electrical block of varying severity presents the most common conduction disorder in LC. Although usually mild, AV block can progress rapidly from a prolonged PR-interval to a His-Purkinje block within minutes to hours and days. Other less common manifestations of LC include endocarditis, myocarditis, pericarditis, dilated cardiomyopathy, and heart failure [5].

AV block secondary to LC generally lasts about three to 42 days, with complete heart block usually resolving within one week. Those with higher degrees of AV block were more likely to be symptomatic [6]. Temporary cardiac pacing is frequently needed by patients who have severe heart block with hemodynamic instability. Overall prognosis is good given that LC typically resolves with antibiotic treatment alone and cardiac intervention is often not needed [7].

Our case highlights the importance of ruling out reversible causes such as electrolytes disturbance, medication causes (our patient had normal electrolytes level, no history of obstructive sleep apnea). LC should be on the differential and appropriate workup was done when a patient presents with a heart block, especially in an endemic area. Our patient was appropriately treated with antibiotics without the need for pacemaker implantation. Upon cardiology evaluation at outpatient, our patient had resolution of his heart block, and no further intervention was needed.

## Conclusions

Lyme disease is a multisystem disorder that can present with cardiac features. Lyme carditis can be the initial presentation, typically one to two months after the onset of infection. Lyme carditis is an important reversible cause of heart block, especially in endemic areas. Prompt recognition of this potentially lethal condition, with appropriate initiation of antibiotics, can improve clinical outcomes and avoid unnecessary pacemaker implantation.
